# New-onset minimal change disease following the Moderna COVID-19 vaccine

**DOI:** 10.1136/bcr-2023-255144

**Published:** 2023-09-15

**Authors:** Nobuteru Kobayashi, Hajime Fujisawa, Jiro Kumagai, Madoka Tanabe

**Affiliations:** 1 Nephrology, Yokohama City Minato Red Cross Hospital, Yokohama, Japan; 2 Pathology, Yokohama City Minato Red Cross Hospital, Yokohama, UK

**Keywords:** COVID-19, Nephrotic syndrome

## Abstract

We report the case of nephrotic syndrome after COVID-19 vaccination. The patient was a man in his 30s with no comorbidities other than atopic dermatitis. Over the course of 2 weeks after the first COVID-19 vaccination, systemic oedema gradually appeared. He was referred to the nephrology department for investigation of the systemic oedema. On admission, he presented with pitting oedema in his lower extremities. Initial examinations revealed massive urinary protein and decreased serum albumin, at 13.9 g/g Cr and 1.5 g/dL, respectively. Renal biopsy was performed, and minimal change disease was diagnosed. Prednisolone 60 mg/day was promptly started on the 5th day of hospitalisation, and complete remission was achieved on the 12th day. Prednisolone was once tapered off in 1.5 years successfully though minimal change disease was relapsed in 1 month after the steroid withdrawal.

## Background

SARS-CoV-2 infection and the resulting COVID-19 have caused extensive morbidity and mortality worldwide in the last 3 years.^1^ Therefore, safe and effective prophylactic vaccines are urgently needed to limit contagion and mortality. Currently, various COVID-19 vaccines are being administered worldwide, which show preventive effects for COVID-19 infection. Most reported side effects of the vaccines are mild or of moderate severity (ie, do not prevent daily activities) and are limited to the first 2 days after vaccination.^2–4^ Major side effects of these vaccines are uncommon.

New-onset minimal change disease (MCD) requiring treatment after COVID-19 vaccination has been reported previously. MCD is the leakage of large amounts of protein into the urine due to increased permeability of the glomerular filtration barrier. The involvement of vaccination in the onset and recurrence of MCD has been known, and most cases develop within 2 weeks after vaccination. Thirty cases of new-onset MCD after COVID-19 vaccination have been reported worldwide, including 16 cases related to the Pfizer-BioNTech vaccine, 5 cases related to the Moderna vaccine, 5 cases related to the AstraZeneca vaccine, 2 cases related to the Sinovac vaccine and 2 cases related to the Janssen vaccine.^5–30^ Only two full case reports of new-onset MCD following the Moderna messenger RNA (mRNA)-1273 SARS-CoV-2 vaccine have been reported.^12 29^ In addition, both reports describe short-term treatment courses.

In this case report, we present a case of new-onset MCD following the first injection of the Moderna mRNA-1273 SARS-CoV-2 vaccine. We successfully maintained remission with steroid monotherapy and completely terminated steroids after 1.5 years. We report our findings with pathology and literature review.

## Case presentation

The patient was a man in his 30s with a history of atopic dermatitis and obesity with a body mass index of 31 kg/m^2^. The day after the patient received the first dose of the Moderna mRNA-1273 SARS-CoV-2 vaccine, he experienced low-grade fever and swelling at the site of the injection. The fever and local swelling improved after 1 day, but leg oedema appeared 2 days after vaccination. Over the course of 2 weeks, generalised oedema, palpitations and shortness of breath gradually worsened. He visited a local doctor and was prescribed a diuretic, but the symptoms did not improve. Therefore, 15 days after the vaccination, the patient was referred to our department and was admitted that day.

On admission, his blood pressure was 142/93 mm Hg and heart rate was 81 beats per minute. His body weight had increased from 90 kg to 101 kg with systemic oedema. Blood tests revealed serum creatinine 0.85 mg/dL, blood urea nitrogen 17.2 mg/dL, serum albumin 1.5 g/dL, Low Density Lipoprotein (LDL) cholesterol 363 mg/dL. In addition, a high degree of proteinuria with urinary protein-creatinine ratio (UPCR) of 13.9 g/g Cr was observed, meeting the diagnostic criteria for nephrotic syndrome. A 24-hour urinary collection revealed proteinuria of 18 346 mg/day, and the selectivity index of urinary protein was 0.094. Antibody screening tests such as antinuclear antibody, antineutrophil cytoplasmic antibody and antiglomerular basement membrane were all negative. Total haemolytic complement (CH 50) and the serum complement levels of C3 and C4 were within normal range. Serum immunoglobulin (Ig) E level was markedly elevated at 2620 IU/mL. A PCR test for SARS-CoV-2 was negative. However, the SARS-CoV-2 anti-S antibody titre was 452 U/mL using the Elecsys Anti-SARS-CoV-2 S RUO (Roche Diagnostics K.K.) and 2650.3 AU/mL using the ARCHITECT SARS-CoV-2 IgG II Quant (Abbott Japan), which was markedly elevated. CT examination showed normal kidney size and ascites effusion.

## Investigations

**Figure 1:**
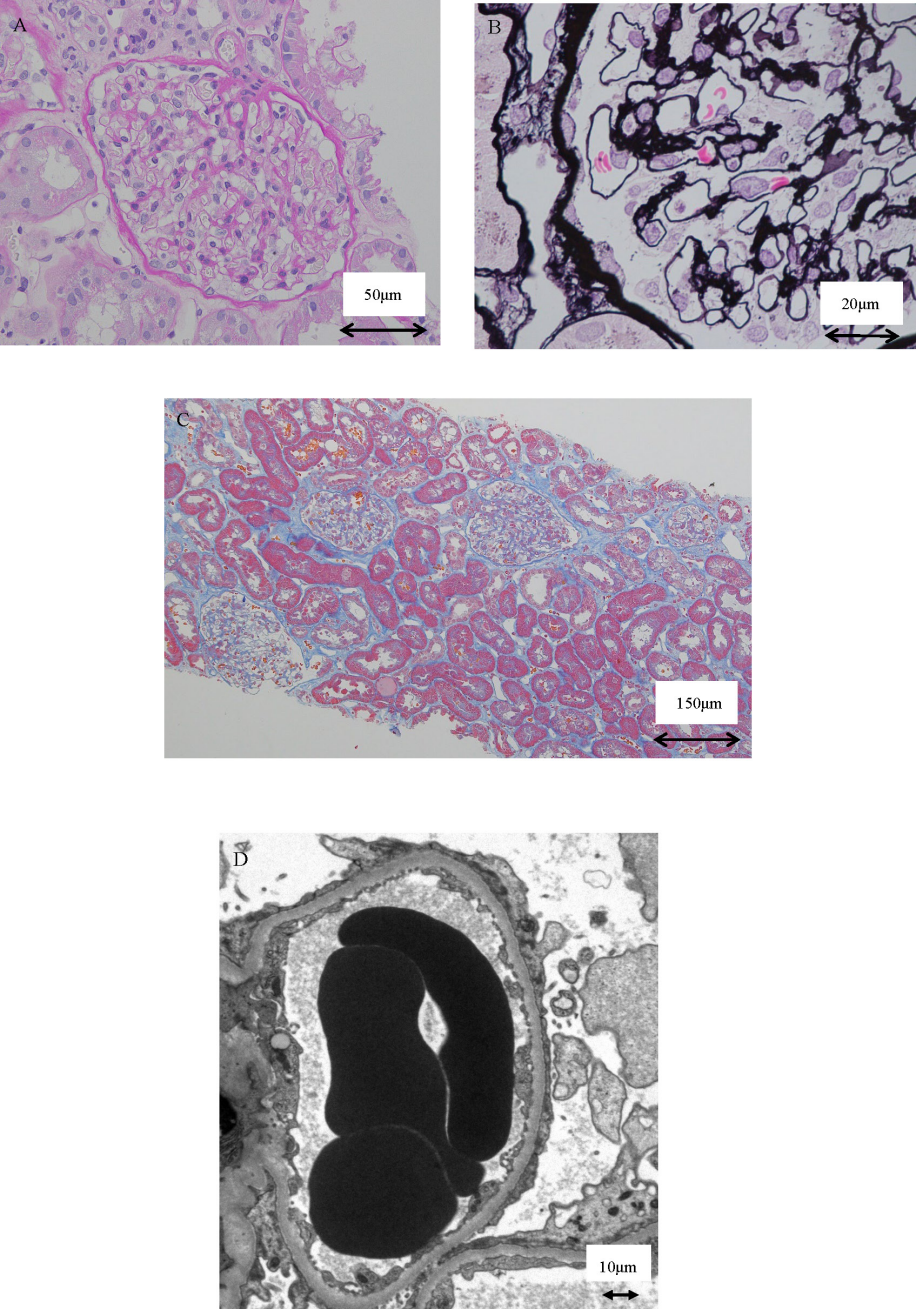
Renal biopsy findings. (A) Normal appearance glomeruli (H&E stain; ×400). (B) Glomerular basement membrane of normal thickness with no deposits (periodic acid methenamine silver stain; ×1000). (C) No evidence of tubular injury, tubular atrophy or interstitial fibrosis (masson trichrome stain; ×100). (D) Extensive foot process effacement of podocytes was observed (electron microscopy; ×4000).

Renal biopsy was performed the day after admission. The histological findings are shown in figure 1. Twenty-three glomeruli were detected, one exhibited global sclerosis and the others were normal. Immunofluorescence staining for IgG, IgA, IgM, C3, C4, C1q and fibrinogen were all negative. Electron microscopy showed normal thickness of the glomerular basement membrane and diffuse foot process effacement. These pathological findings were compatible with MCD.

## Treatment

**Figure 2:**
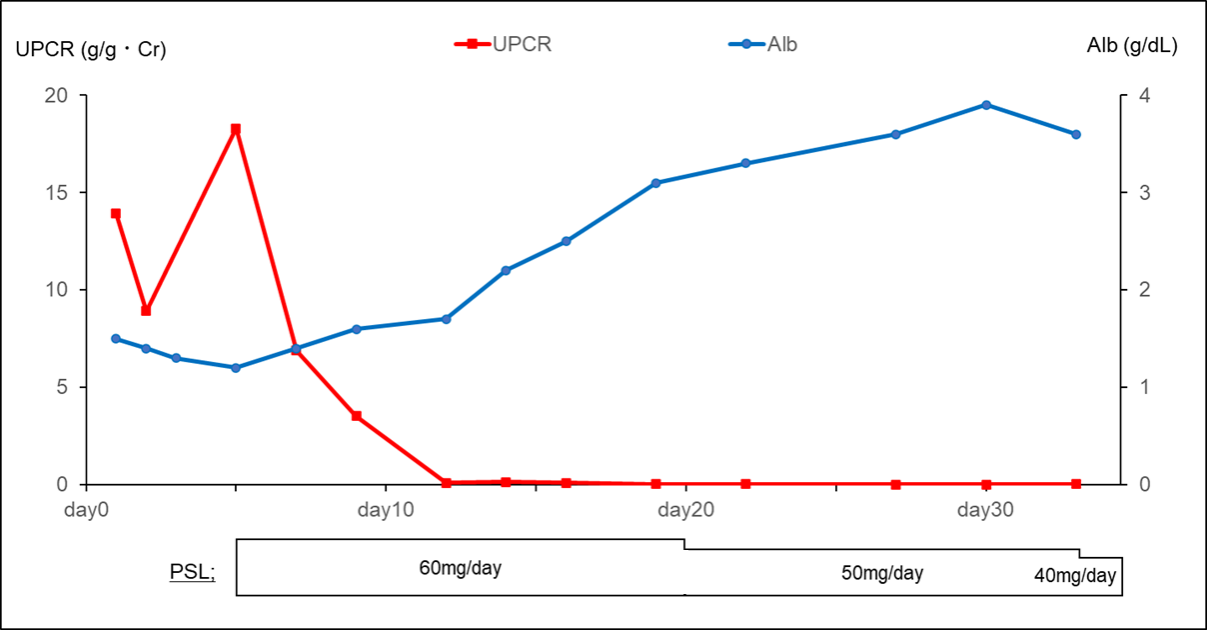
Clinical course and treatment of the patient. Alb, serum albumin; PSL, prednisone; UPCR, urinary protein-creatinine ratio.

The clinical course of this case is shown in figure 2. Prednisolone (PSL) 60 mg/day was started on the 5th day of hospitalisation.

## Outcome and follow-up

On the 12th day, the UPCR decreased to 0.1 g/g Cr and complete remission was achieved. After the remission, time was required to manage the side effects of high-dose steroids (eg, blood glucose control and osteoporosis prevention), and the patient was discharged on day 34 of hospitalisation when the dose was reduced to moderate-dose steroids. Two months after discharge, serum creatinine was 0.62 mg/dL, serum albumin 4.6 g/dL and UPCR was 0.14 g/g Cr. The patient remained in complete remission for more than 1 year and PSL was completely tapered off approximately 1.5 years after the onset. However, 1 month after completion of PSL treatment, a relapse of nephrotic syndrome was observed, so PSL 20 mg was restarted, and remission induction was achieved quickly. The second vaccination was not administered based on consultation with the patient. We suggested that if the patient wanted to be vaccinated in the future, another type of COVID-19 vaccine, such as inactivated vaccine, could be administered, but the patient did not wish to be vaccinated.

## Discussion

This case highlights the risk of new-onset MCD after mRNA vaccine administration. Several case reports in regard to the association of MCD with the Moderna mRNA-1273 SARS-CoV-2 vaccine have been published previously. Full case reports of new-onset MCD cases after Moderna mRNA-1273 SARS-CoV-2 vaccination are few and no reports mention long-term outcomes. In MCD, a state of hyperpermeability of glomerular capillaries occurs, resulting in highly selective proteinuria. Although the exact pathogenesis has not yet been elucidated, injury of podocytes by circulating factors released by T cells is thought to be the main mechanism.^31 32^ This case highlights the rare but potential risk of MCD after mRNA vaccine administration. There are several case reports of new-onset MCD after mRNA vaccination, and the chronological causality is convincing.^5–30^ Although the mechanism is unclear, this case raises the question of whether the mRNA SARS-CoV-2 vaccine may lead to rapid development of MCD within a few days following vaccination.

The inevitable question is whether the timing of COVID-19 vaccination and the onset of nephrotic syndrome were too close together. The timing of MCD onset or relapse was reported at a median of 7 days following the first dose of the COVID-19 vaccine.^33^ On the other hand, four cases of new-onset MCD within 1–2 days following COVID-19 vaccination have been reported.^9 14 15 27^ Therefore, the COVID-19 vaccine is estimated to have the potential to trigger an immune response that leads to the development of MCD within 2 days. In addition, the SARS-CoV-2 anti-S antibody titre was markedly elevated in this case, suggesting hypersensitivity to the mRNA vaccine. The antibody titre, measured using the Roche test after the first vaccination, was reported to be about 100 U/mL in a population without previous infection; the titre was 452 U/mL in this case, more than four times higher.^34^ In comparison, the antibody titre in a previously infected population was reported to be about 10 000 U/mL.^34^ In this case, SARS-CoV-2 anti-S antibody titre is in an intermediate range and previous subclinical infection with SARS-CoV-2 cannot be ruled out completely. This finding may explain the brief time between vaccination and onset of MCD.

In combination with COVID-19 vaccination, atopic disease may have predisposed this patient to developing MCD. Pre-existing atopic dermatitis has been associated with the development of MCD through IgE-mediated hypersensitivity, which could imply that those with atopic dermatitis become more susceptible to potential allergic stimulation such as mRNA vaccination, leading to MCD, than those without atopic dermatitis. Additionally, MCD is frequently observed in patients with bronchial asthma and atopic dermatitis, and the relationship between IgE-mediated type I allergy and MCD has been highlighted.^35–37^ In this case, the background disease was atopic dermatitis, and the blood test at the time of admission showed high IgE levels, indicating that the patient was predisposed to hypersensitivity and developing MCD following vaccination. Similarly, Salem *et al* reported a case of new-onset MCD following the Pfizer-BioNTech SARS-CoV-2 vaccine in a 4os woman with asthma.^11^ In this case report, as in the present case, a short period of 5 days was observed between mRNA vaccination and the onset of MCD. Accumulation of cases in the future may suggest an association between atopic disease and MCD after mRNA vaccination.

Steroid sensitivity of new-onset MCD after mRNA vaccination is often good, and there are no case reports of spontaneous remission. Most previously reported cases without underlying disease describe remission induced with steroids alone.^5–30^ In this case, complete remission was quickly achieved with PSL 60 mg/day (1 mg/kg), and the steroid response was particularly good. However, a relapse of the nephrotic syndrome was observed soon after the steroid treatment was terminated, so careful follow-up is necessary. Based on case reports to date, early detection of nephrotic syndrome after COVID-19 vaccination and therapeutic intervention are important factors. Nephrologists and allergy specialists involved in vaccination need to be well informed of the possibility of side effects and ensure that patients receive adequate follow-up after vaccination. COVID-19 vaccine is an effective vaccine that can significantly reduce infection. This case report is not to deny vaccination, but to show the necessity of proceeding with vaccination after being aware of the possible side effects. The limitation in this case is the coincidental temporal association. In other words, we cannot deny the possibility that the vaccination and the onset of MCD were unrelated and occurred by chance. More studies are needed to elucidate the relationship between MCD and mRNA vaccines.

**Learning points**

- Although the causal relationship is uncertain, minimal change disease following mRNA vaccination has been reported.
- As a possible pathogenic mechanism, the mRNA vaccination may have affected the immune regulatory system, especially the T-cell profile, causing podocyte damage.
- In addition, the patient had atopic dermatitis as a background disease, and it is thought that the predisposing factor was an atopic disease characterised by a Th2-dominant immune state.
